# Ability-Based Emotional Intelligence Is Associated With Greater Cardiac Vagal Control and Reactivity

**DOI:** 10.3389/fnhum.2019.00181

**Published:** 2019-06-11

**Authors:** John R. Vanuk, Anna Alkozei, Adam C. Raikes, John J. B. Allen, William D. S. Killgore

**Affiliations:** ^1^Social, Cognitive, and Affective Neuroscience Laboratory, Department of Psychiatry, University of Arizona, Tucson, AZ, United States; ^2^Psychophysiology Laboratory, Department of Psychology, University of Arizona, Tucson, AZ, United States

**Keywords:** cardiac vagal control, emotional intelligence, mixed emotional intelligence, ability emotional intelligence, heart rate variability, stress, autonomic control, emotion regulation

## Abstract

Several distinct models of emotional intelligence (EI) have been developed over the past two decades. The ability model conceptualizes EI as a narrow set of interconnected, objectively measured, cognitive-emotional abilities, including the ability to perceive, manage, facilitate, and understand the emotions of the self and others. By contrast, trait or mixed models focus on subjective ratings of emotional/social competencies. Theoretically, EI is associated with neurobiological processes involved in emotional regulation and reactivity. The neurovisceral integration (NVI) model proposes a positive relationship between cardiac vagal control (CVC) and cognitive-emotional abilities similar to those encompassed by EI. The current study examined the association between CVC and EI. Because ability EI is directly tied to actual performance on emotional tasks, we hypothesized that individuals with higher ability-based EI scores would show greater levels of CVC at rest, and in response to a stressful task. Because mixed-models of EI are not linked directly to observable emotional behavior, we predicted no association with CVC. Consistent with expectations, individuals with higher levels of ability EI, but not mixed EI, had higher levels of CVC. We also found that individuals with greater levels of CVC who demonstrated reactivity to a stress induction had significantly higher EI compared to individuals that did not respond to the stress induction. Our findings support the theoretically expected overlap between constructs within the NVI model and ability EI model, however, the observed effect size was small, and the associations between EI and CVC should not be taken to indicate a causal connection. Results suggest that variance in the ability to understand emotional processes in oneself and to reason about one’s visceral experience may facilitate better CVC. Future work manipulating either CVC or EI may prove informative in teasing apart the causal role driving their observed relationship.

## Introduction

Emotion and cognition represent a dynamic system involved in a reciprocal relationship. Their interplay is proposed to facilitate social adaptation, thus facilitating the optimization of health, and behavior (Lazarus, 1991). The facilitation and balance between emotion and cognition are thought to be a unique set of abilities, separate from general intelligence (IQ), and conceptualized as emotional intelligence (EI) (Salovey and Mayer, 1990). EI has been defined in a number of ways but is generically described as the awareness and understanding of emotional information relating to oneself and others, and the ability to use that information to facilitate goal-oriented behavior (Payne, 1985; Mayer and Geher, 1996; Goleman, 2006; Smith et al., 2018). The concept of EI has been defined by multiple authors, to index the degree to which emotion relates to adaptive human behavior and social engagement (Bar-On, 2004; Extremera and Fernández-Berrocal, 2005; Pérez et al., 2005; Seal et al., 2009).

Two main models for quantifying EI for research purposes have emerged from previous work and are proposed as either purely “performance ability” or “trait” based (Bar-On, 1997; Mayer et al., 2008). The ability model postulates EI as a narrow construct and uses objective performance-based testing as a means of quantification, whereas trait models theorize that it is a broader skill set and have become known as mixed-models of EI (Mayer et al., 2002; Bar-On, 2004; Gutiérrez-Cobo et al., 2016). The Mayer-Salovey-Caruso Emotional Intelligence Test (MSCEIT) is the most widely used performance-based ability model test and encompasses a variety of skills and abilities related to emotional processing, such as the ability to use emotions purposefully, distinguish emotional cues, and deliberately use emotions when having to make decisions (Mayer et al., 2001; Brackett et al., 2006). The model proposes a hierarchy of performance based on the quality of responses to a variety of emotion-focused problems. As an ability measure, the MSCEIT comprises a series of tasks that assess discrete emotional skills such as the ability to recognize emotions in visual stimuli such as faces and photographs, the ability to regulate and manage emotions in various contexts, and the ability to solve emotional problems. On the other hand, the Bar-On emotional quotient inventory (EQ-i) is one of the most widely used self-report instruments assessing mixed model EI (Bar-On, 2004). The Bar-On model, and other mixed approaches, generally conceive of EI as a set of stable social and emotional competencies of which the individual is self-aware and that are assessed via self-report statements (Petrides and Furnham, 2001). An alternative conceptualization of EI that has gained attention for its potential to foster a more holistic theory of EI calls for a multi-level model and the integration of a behavioral level of EI relating it to social and professional outcomes (Boyatzis, 2018). A multi-level approach will undoubtedly propel our understanding of EI as a unique and specific construct relating to cognition and behavior. However, the time and resource demands of the qualitative methodology inherent to this type of assessment of EI capacities has been a barrier to its larger scale dissemination and application in research. As such, practitioners wishing to assess EI find themselves having to consider not only theoretical orientations but the resources necessary to adequately assess the capacity (Boyatzis, 2018). Despite more than two decades of intensive research, there continues to be a wide-ranging debate about the construct validity of the various models of EI and how such models relate to cognition (Mayer et al., 2001; Locke, 2005; Alkozei et al., 2018).

It is well accepted that effective emotion regulation strategies contribute to wellbeing and positive mental health outcomes. Higher levels of EI are particularly strong predictors of mental health outcomes and also associated with better physical health (Martins et al., 2010; Fernández-Abascal and Martín-Díaz, 2015). Interestingly, mixed and ability measures of EI are often poorly correlated with one another and predict different aspects of health (Webb et al., 2013). For example, higher mixed EI has been associated with increased well-being, yet attempts to replicate this association with ability tests have been unsuccessful (Furnham and Petrides, 2003; Zeidner and Olnick-Shemesh, 2010). Higher levels of mixed EI may also protect against emotion dysregulation and facilitate greater quality in social interactions (Lopes et al., 2005). Individuals with higher levels of mixed EI demonstrate increased resilience against decrements in personally relevant moral-judgment decisions while sleep deprived, without significant changes for other moral domains (Killgore et al., 2007). Both mixed and ability model scores demonstrate a positive association with accuracy in recognizing emotional facial expressions (Petrides and Furnham, 2003; Wojciechowski et al., 2014). There is a high correlation between mixed EI and job performance, however, well-established measures of knowledge, skills, abilities, and other characteristics offer significant incremental prediction beyond measures of mixed EI (Joseph et al., 2015). The mixed findings for the two models underscore the notion that associations between EI as a quantified measure are dependent on several factors, including cognition, the testing modality, and the degree of emotional content inherent to the endeavor. A growing body of work demonstrates a distinction between cognitive tasks that are emotionally neutral, as opposed to those containing affective stimuli, conceptualized as utilizing “hot” or “cool” cognitive processes (Metcalfe and Mischel, 1999). A recent systematic review of studies showed that ability, but not mixed, EI correlated positively with task performance that required “hot” cognitive processes, while studies investigating the relationship between EI metrics, and “cool” cognitive processes failed to produce any positive associations (Gutiérrez-Cobo et al., 2016). Based on their findings, the authors concluded that current ability and mixed model conceptualizations of EI are only relevant for tasks that require affective processing and that the MSCEIT is the only current ability or mixed model based EI measure that reliably predicts increased performance on affective cognitive tasks.

Emotional functioning depends on a dynamic interplay of the central nervous system and the autonomic nervous system. A fundamental component of the parasympathetic branch of the autonomic nervous system critical to mind-body interactions is cardiac vagal control (CVC), which provides a reliable marker for emotional health (Porges, 1995). Measures of CVC, such as heart rate variability (HRV), are considered representative of interindividual differences in parasympathetic efferent control of cardiac rate, which when high promotes adaptive emotional responding and regulation that underlie physical, and mental health (van Ravenswaaij-Arts et al., 1993; Acharya et al., 2007; Beauchaine and Thayer, 2015). Optimal cardiac reactivity demonstrates tightly coupled reciprocal responsiveness between the sympathetic and parasympathetic systems in reaction to environmental demands (Mccabe et al., 2000). The Polyvagal theory posits that CVC is responsible for higher-order functions in mammals, from a phylogenetic perspective, by facilitating emotion regulation, and promoting social engagement (Porges, 1995). Individual differences in CVC reactivity predict vulnerability to stress, along with positive outcomes in communication, attention, and the regulation of emotion (Suess et al., 1994; Porges, 1995; Thayer and Lane, 2000; Appelhans and Luecken, 2006). A substantial body of work demonstrates associations between low levels of resting and reactive CVC with multiple forms of internalizing and externalizing psychopathology (Kemp and Quintana, 2013; Shahrestani et al., 2014; Beauchaine, 2015). Greater CVC reactivity buffers against the development of psychopathology and health problems (El-Sheikh et al., 2001). Increases in CVC are related to optimal outcomes in the treatment of major depression and have been suggested as a target for anxiety interventions (Chambers and Allen, 2002; Chalmers et al., 2014).

Cardiac vagal control is important in a wide range of situations that demand effective cognitive-emotional regulation through coordination of biological systems. The degree of influence CVC has over the central autonomic network is thought to rely on prefrontal inhibition, and the neurovisceral integration (NVI) model proposes that the dynamic system contributing to autonomic control involves a negative feedback system guided by attention regulation and affective processing (Thayer and Lane, 2000). The central autonomic network demonstrates a positive relationship between CVC modulation and increased cognitive-emotional abilities that are similar to EI and is predictive of positive behavioral health outcomes in mood disorders, such as depression (Friedman, 2007). Since its initial conception, the NVI model has gained considerable empirical support relevant to positive behavioral health outcomes, and recent work has extended NVI model to a hierarchical model involved in predictive cognitive coding computations (Smith et al., 2017). Cognitive coding computations are critical during early development and are vital to learning and the comprehension of knowledge (Clark and Paivio, 1991). Children with higher levels of resting HRV have higher capacities for sustained attention and higher attention span/persistence in early childhood; which contribute to cognitive development and higher educational attainment (Suess et al., 1994; McClelland et al., 2013). Increased performance in high-stress environments such as sports competition is also linked to greater ability to regulate emotions, as well as resting CVC and its modulation (Crombie et al., 2009; Plews et al., 2012). The use of biofeedback to enhance CVC control is related to improvements in emotional health in the workplace as well as better performance in competitive environments where high levels of stress are inherent (McCraty et al., 2003; Holden, 2006). Favorable results related to increases in resting CVC recovery following a stressor after targeted biofeedback training have been shown to occur and contribute to positive outcomes related to anxiety and emotion regulation (McCraty et al., 1999; Thurber et al., 2010).

Chronic stress and the inability to regulate emotions are associated with maladaptive physiology, mental health, and have a critical impact on multiple aspects of well-being (Chrousos, 2000). Higher levels of ability-based EI predict greater CVC reactivity during more intense emotional experiences (Rash and Prkachin, 2013). Self-report mixed EI metrics also predict positive outcomes and better cardiac responsiveness during stress (Bar-On et al., 2006). Athletes with higher levels of mixed EI have lower levels of CVC reactivity during high-stress competitive environments (Laborde et al., 2011). However, this must be considered in light of the fact that athletes demonstrate atypical autonomic reactivity compared to less physically fit individuals and have significantly higher levels of self-esteem and social connectedness (Koivula et al., 2002; Plews et al., 2012). In conclusion, studies investigating the relationship between the different conceptualizations of EI, CVC, and its reactivity to stress demonstrate consistent positive associations between EI and CVC; but no study to date has investigated this relationship incorporating two of the most widely used and validated measures of mixed and ability EI simultaneously.

To address the current gap in literature associating CVC and EI, we examined the influence of both ability EI and mixed EI on CVC during rest and in response to a potentially stressful task. Since CVC reactivity is linked to flexibility in emotion regulation and ability-based metrics are most representative of cognitive control, likely extending to EI, then individual differences in EI are expected to be related to CVC modulation during stress. In light of prior evidence, three general hypotheses were tested. First, we hypothesized that individuals with higher levels of ability EI would have greater levels of CVC at rest, and if so by the perceiving and understanding domains that are less likely to incorporate acute CVC reactivity. Second, we hypothesized that individuals with higher levels of ability EI would have greater decreases in CVC in response to stress and greater subsequent increases during recovery, and if so, these would be driven by the managing and using domains, which may be more likely to be utilized in contexts requiring CVC reactivity. Finally, we hypothesized that cardiac metrics that are less specifically sensitive to vagally mediated influences (i.e., sensitive to some extent also to sympathetic influences) would not demonstrate the same associations with EI as CVC metrics that reflect primarily parasympathetic influence.

## Materials and Methods

### Participants

One hundred thirty-five healthy adults (87 females) were recruited from the local community via internet, newspaper, radio, and flyer advertisements for the present study. A power analysis modeled on previous work investigating CVC and emotional dispositions suggested that effect sizes were small to medium (Pearson’s *r* ranged from 0.21 to 0.38) (Oveis et al., 2009). Therefore, for the proposed study, we applied the mean effect size (*r =* 0.29) to estimate power to detect individual differences. That power analysis showed that with α = 0.05 (2-tailed), a sample of *n* = 88 should provide adequate power (1-β = 0.8) to detect individual differences to be established by CVC characteristics, which was less than the minimum number of individuals to be recruited for a subsequent study sharing a recruitment effort with the present study. Participants were between the ages of 18–40. Because of the high reading and cognitive demands of the tasks, participants were required to have an English reading proficiency of 8th grade or higher as defined by the WRAT4 Reading subtest and were also screened to exclude individuals with impaired reading comprehension, and altered mental status or capacity, due to medications, substances, cognitive status, injury, or medical conditions that could influence the outcome of psychological assessment.

Participants were excluded from analyses if they took medication with a mechanism of action that influences cardiac reactivity (21 participants, see Supplementary Appendix A for a list of medications) or had unusable EKG data (8 participants). Four participants failed to have their EQi recorded and were also removed from analyses. The final sample for analyses included 102 individuals (64 females, mean age = 22.8 years, *SD* = 4.4). All participants had a high school diploma or equivalent, 91.2% of participants completed some college, 28.4% of participants had a Bachelor’s degree or higher. 54.9% of participants were Caucasian, 21.6% were Hispanic/Latino, 12.7% were Asian/Pacific Islander, 4.9% were African American, and 5.9% reported ethnicity as “other.”

All participants provided written informed consent before enrollment. The study protocol was approved by the Institutional Review Boards of the University of Arizona and the U.S. Army Human Research Protections Office.

### Apparatus and Materials

#### Psychological Assessment

The Mayer-Salovey-Caruso Emotional Intelligence Test II (MSCEIT) was used to assess ability-based EI and evaluates a number of specific skills and abilities related to reasoning about and regulating emotional processes (Mayer et al., 2003). The MSCEIT is a 141-item performance test requiring subjects to identify emotions in faces and designs, to specify emotions or feelings that interfere with or facilitate specific thought processes, demonstrate an understanding of how various emotions combine to create higher-order emotions and how these blends may change over time, as well as demonstrate knowledge of how specific emotional management strategies will lead to various consequences in oneself and others. The test yields a total EI score, two area scores (experiencing and strategic), and four branch scores (perceiving, using, understanding, and managing); derived from eight task-level scores. The area scores are specific to the ability to recognize emotions and determine how they interact with a thought or understand emotional meanings relative to others and manage them. The branch scores are specific to the ability to identify emotions, facilitate thought using emotions, understand emotions, and manage emotions. The MSCEIT has been found to have adequate reliability (split-half reliability overall = 0.91) and good discriminant and convergent validity (Mayer et al., 2002).

Mixed EI was assessed using the *Bar-On EQ-I 2* (EQi), a self-report inventory designed to evaluate the construct of EI and the underlying factors that contribute to emotionally intelligent behavior (Bar-On et al., 2006). The EQi is a 133-item self-report measure using short sentences (e.g., “I am good at reading other people’s emotions”) and a 5-point Likert response scale ranging from (1) “very seldom or not true of me” to (5) “very often true of me or true of me.” The measure provides several scores, including a general metric of total EI and five composite scales (self-perception, self-expression, interpersonal, decision making, stress management), assessing various features of mixed EI. The EQi has been found to have good discriminant and convergent validity, as well as very high reliability (internal consistency = 0.97) (Bar-On, 2004).

#### Physiological Assessment

Physiological data were recorded using a Zephyr Biopatch^1^ with conductive adhesive hydrogel foam electrodes. The device was placed at the sternum, and the ECG signal was sampled at 1000 Hz, which is above the minimum suggested sampling frequency (Camm et al., 1996). Off-line analysis was performed by extracting the inter-beat interval (IBI) series from the raw digitized ECG signal using QRSTool Software (Allen et al., 2007).

### Procedure

After providing informed consent, participants underwent an ECG reactivity assessment that entailed two five-minute resting periods separated by a 90-s cognitive challenge as a stress induction (serial subtraction) (Tomaka et al., 1994; Seraganian et al., 1997). During resting periods, participants were instructed to sit quietly without talking or moving while focusing on a fixation cross positioned in front of them. During the stress induction, participants were asked to count backward by 17, starting from 1,025, as quickly as possible. Participants were provided pre-recorded auditory feedback contingent on their performance via an E-Prime program^2^, controlled by a research technician. To maximize the stress induction, participants were instructed to begin again if they made an error in their subtraction, or that they needed to go faster and to start again if they reached predetermined points without error. Participants completed all psychological measures, including the MSCEIT and EQi, subsequent to the ECG reactivity assessment.

#### Physiological Data Reduction and Variable Selection

The extracted IBI series were hand corrected by a trained and experienced technician to eliminate artifacts such as ectopic, erroneous, and missed beats (Berntson et al., 1990). Data were processed using Matlab (version 2015B^3^) with parameters modeled on those of CMetX Cardiac Metric Software (Allen et al., 2007), with the additional incorporation of a moving window. The moving window used 30-s segments that shifted by 3-s at a time. Estimates of multiple metrics of cardiac chronotropy were derived using the extracted time series. The moving window approach ensures that variance estimates from a non-stationary time series (as is almost always the case with interbeat-interval data) are not inflated by recording length. The mean value across all 30-s epochs was taken for each metric as the final value to be used in analyses.

#### CVC Variable Selection and Estimates

Increased influence of the vagus nerve on heart rate leads to larger variance in the time interval between heartbeats; a phenomenon classified HRV. HRV is widely accepted as representative of CVC and is sensitive to both the parasympathetic and sympathetic influences of the autonomic nervous system, but it reflects predominantly parasympathetic influences when individuals are at rest (Allen et al., 2007; Kromenacker et al., 2018). The root mean square of successive differences (RMSSD) is another time-domain measure proposed to quantify the parasympathetic nervous system’s impact on HRV (Von Neumann, 1941; Malik et al., 1996). The Polyvagal theory proposes respiratory sinus arrhythmia (RSA) as a measure of CVC, indexing the magnitude of respiratory-linked changes in HRV (Porges, 1995). Individual differences in RMSSD are associated with similar outcomes as RSA, such as susceptibility to cardiovascular disease, but more sensitive to low-frequency HRV contributions that can represent a combination of parasympathetic and sympathetic influences on HRV (Berntson et al., 2005; Thayer et al., 2010; Kromenacker et al., 2018). While RSA is the gold standard in the non-invasive quantification of parasympathetic control, it is still an imperfect index of CVC, as it is susceptible to the influence of respiration (Grossman et al., 1991; Allen et al., 2007). RMSSD is less sensitive to the impact of slow respiration and individuals breathing outside the targeted range, but debate remains regarding the underlying influence on this time domain metric (Hill et al., 2009). Previous work shows high correlations between RSA and RMSSD (*r*’s = 0.85–0.91*)* but RSA has higher correlations with HRV (*r =* 0.90) than RMSSD (*r* = 0.84) (Berntson et al., 2005; Kleiger et al., 2005; Allen et al., 2007).

Respiratory sinus arrhythmia was estimated by calculating HRV in the high-frequency band that captures respiratory-related changes in the timing of heart beats (0.12–0.4 Hz). The IBI series was converted to a time series sampled at 10 Hz with linear interpolation and a 241-point optimal finite impulse response digital filter designed using FWTGEN V3.8 (Cook and Miller, 1992) with half amplitude frequencies of 0.12–0.4 Hz. RSA is the natural log of the variance of this filtered time series. HRV is the natural log of the variance of the unfiltered time series. RMSSD was also quantified as a measure of CVC, to be utilized in the event the RSA metric was compromised by individuals with a peak frequency of respiration below 0.12 Hz. An estimate of RMSSD was derived using the root mean square of successive differences in the IBI time series across each moving window. An index of respiration rate was obtained using a fast Fourier transform on the IBI series, and the dominant frequency in the power spectrum of the respiration waveform was inspected to ensure the rate did not fall below 0.12 Hz, which invalidates the estimate of RSA for such segments (Grossman and Taylor, 2007). During the resting baseline period, 77.2% of individuals had max power frequencies that fell below 0.12 Hz. Due to this problem, which invalidated the RSA measure in the majority of subjects, all analyses targeting CVC were conducted using RMSSD rather than RSA. CVC estimates represent mean levels during each unique condition (i.e., during the baseline resting period, during the stressor, and during the post-stress recovery resting period).

#### MSCEIT Scoring

Raw data from the MSCEIT were scored by the Multi-Health-Systems using consensus scoring adjusted for age and gender. Consensus scoring is based on the concept that general consensus should identify the optimal answer to the majority of emotion-based questions, as emotions are evolved signals that require the majority of the group to understand and accept as valid/accurate (Mayer et al., 2003).

#### Statistical Analysis

All statistical tests used an *a priori* significance level of *p* ≤ 0.05. Inspection of skewness, kurtosis, and the Shapiro-Wilk test indicated non-normal distributions among multiple variables. Due to deviations from normality and the presence of heteroscedasticity, log transformations of variables of interest were employed. However, as some variables of interest still failed to achieve normality assumptions, optimal model parameters were identified using akaike’s information criterion (AIC) penalized-likelihood criteria, and the optimal model parameters reported utilized employed fixed variances and separate covariances with restricted maximum likelihood estimation.

##### Software and packages utilized

All statistical analyses were performed using R (version 3.5.1^4^). Correlation analyses were performed using the *hmisc* package (Harrell, 2018). Linear regression model assumptions were interrogated and ensured to have been met using the *gvlma* package (Pena and Slate, 2014). Linear models were analyzed utilizing the *nlme* and *predictmeans* packages (Luo et al., 2014; Pinheiro et al., 2014). Penalized likelihood criteria were analyzed using the *glmnet* package (Friedman et al., 2010). Hierarchical agglomerative clustering was conducted using the *FactoMineR* package (Lê et al., 2008; Wickham, 2016). Stepwise variable selection was implemented using the stepAIC function in the MASS package (Ripley, 2002). Figures and tables were generated using the *corrplot* and *ggplot2* packages (Wei and Simko, 2013; Lüdecke, 2018).

##### Zero-order correlation analysis

Bivariate Spearman correlations were performed across all subjects for age, total EI scores, and cardiovascular variables for resting baseline, stress reactivity and recovery indices (change in RMSSD from the prior level). EI subscale correlations are presented in the Supplementary Material S3.

##### EI predicting baseline CVC

A simple linear regression model was used to predict baseline cardiac metrics, based on the total score of each EI measure. Outcomes and predictors were log transformed [y’ = log(y)] after the initial model failed to meet the model assumptions of skewness, kurtosis, and heteroscedasticity. Model assumptions were satisfied utilizing the log-transformed variables. To identify which EI subscale drove potential significant effects, a multiple linear regression model was fit using all sub scale scores from the unique EI metric and an automated forward and backward stepwise variable selection method simplified the model to limit multi-collinearity between predictors.

##### Physiological response to serial subtraction

A linear mixed model for repeated measures over time using generalized least squares was used to investigate changes in RMSSD in response to the serial subtraction task and subsequent recovery following the stress induction.

##### EI predicting change in CVC across conditions

The identified optimal parameters for the linear mixed models assessing RMSSD across time were used to investigate the main effect and interactions for EI measures and RMSSD levels across the stress induction and recovery conditions using RMSSD as the dependent variable and condition, total ability EI, and total mixed EI scores as predictors. AIC penalized-likelihood criteria was used to determine if the addition of the EI total scores in a main effect or interaction model had a significant influence beyond the simpler model with only condition (e.g., resting baseline, stress induction, and resting recovery levels), as a predictor.

##### Investigating potential influential covariates

Akaike’s information criterion penalized-likelihood criteria was used to determine if covariates of interest had a significant influence on the models with a least absolute shrinkage and selection operator (LASSO) regression analysis using baseline RMSSD as an outcome variable and gender, age, caffeine consumption that day, and time of day as predictors. Regression models were rerun, accounting for the identified covariates of influence, to investigate potential increases in the total variance accounted for within the models and compared to the simpler model using AIC penalized-likelihood criteria.

##### Exploring individual differences in CVC in response to stress and recovery

To investigate potential group level effects associated with individual differences in CVC, a principal components analysis (PCA) in conjunction with hierarchical agglomerative clustering was employed across RMSSD at rest, stress induction, and recovery. Initial analyses investigating between-group effects were conducted using *t*-tests. However, residuals were not normally distributed, so non-parametric two-tailed Mann-Whitney *U* tests were utilized for group-level analyses examining associations CVC responsiveness to stress induction and EI variables.

##### Secondary analyses

Additional secondary analyses on HRV and HR are presented in the Supplementary Materials, including statistical methods and results. See Supplementary Material S1 and Supplementary Tables S2, S3, S6, S7.

## Results

### Descriptive Statistics and Correlations

Table 1 presents descriptive statistics, means, and associated standard deviations for the demographic, EI, and physiological variables.

**Table 1:** Descriptive statistics of the sample.

Measure	Mean	St. Dev.
Age	22.78	4.39
Baseline RMSSD	32.74	19.64
Stress induction RMSSD	26.81	13.40
Stress recovery RMSSD	33.61	17.94
Baseline HRV	7.26	0.98
Stress induction HRV	7.49	0.84
Stress recovery HRV	7.30	0.90
Baseline HR	84.63	11.15
Stress induction HR	95.20	13.91
Stress recovery HR	83.27	11.48
EQi total	102.79	12.62
EQi interpersonal branch	105.78	13.62
EQi decision making branch	101.85	12.80
EQi stress management branch	104.84	12.30
EQi self perception branch	101.46	13.46
EQi self expression branch	97.64	14.10
MSCEIT total	107.97	12.52
MSCEIT perceiving branch	110.16	12.81
MSCEIT using branch	106.93	13.48
MSCEIT understanding branch	111.84	18.92
MSCEIT managing branch	100.97	11.79


*RMSSD, root mean square of successive differences; HRV, heart rate variability; HR, heart rate; EQi, Bar-On EQ-I 2; MSCEIT, Mayer-Salovey-Caruso Emotional Intelligence Test II; St. Dev., standard deviation.*

### Full Sample Zero-Order Correlations

**FIGURE 1:**
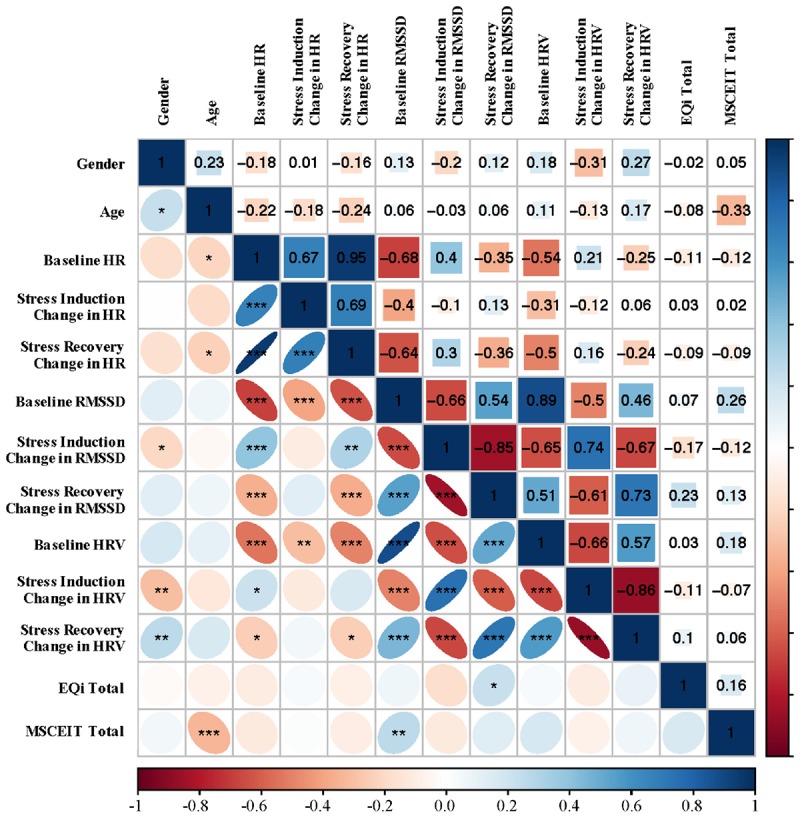
Bivariate Spearman correlations performed across all subjects with correlation coefficients in the upper portion of the matrix and significant correlations identified in the lower portion of the matrix. ^∗^*p* < 0.05, ^∗∗^*p* < 0.01, ^∗∗∗^*p* < 0.001. RMSSD, root mean square of successive differences; HRV, heart rate variability; EQi, Bar-On EQ-I 2; MSCEIT, Mayer-Salovey-Caruso Emotional Intelligence Test II.

Figure 1 presents bivariate correlations assessing relationships among RMSSD, HRV, and HR at baseline resting levels, and stress reactivity and recovery indices (change in RMSSD from the prior level), with age, depression, and total EI scores across the total sample. Baseline RMSSD showed positive associations with the MSCEIT total score. Total mixed EI had a positive association with change in RMSSD from stress induction to recovery. No associations were observed for HRV or HR at baseline, change after stress, or after recovery. Age had a negative association with the MSCEIT total score, as well as baseline and recovery levels of HR; however, age had a high degree of positive skew. None of the observed associations remained significant after Bonferroni correction for multiple comparisons.

### EI Predicting Baseline CVC

#### EI and RMSSD

**FIGURE 2:**
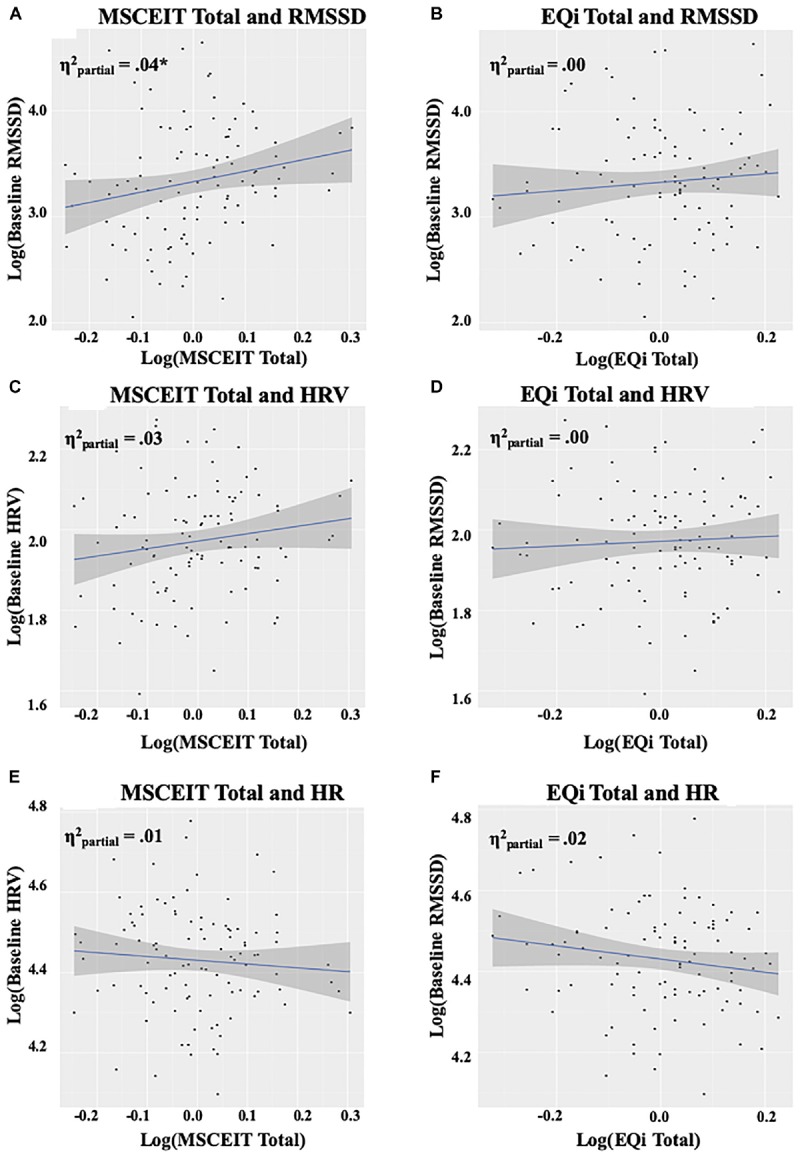
Panels **(A,B)** depict the relationship between EI measures and RMSSD at baseline, panels **(C,D)** depict the relationship between EI measures and HRV at baseline, and panels **(E,F)** depict the relationship between EI measures and HR at baseline. The intercept reflects levels at baseline, and the shaded area represents the 95% confidence interval. Plots and values are displayed on a log scale reflecting the data transformations utilized to meet model assumptions. RMSSD, root mean square of successive differences; HRV, heart rate variability; HR, heart rate; EQi, Bar-On EQ-I 2; MSCEIT, Mayer-Salovey-Caruso Emotional Intelligence Test II; $\eta_{p}^{2}$, partial eta-squared. ^∗^*p* < 0.05.

Total ability EI was a significant predictor for baseline RMSSD, *F*(1,99) = 4.60, *p* = 0.03, (Figure 2A). The observed effect was driven by the understanding branch of ability EI, *F*(1,100) = 3.89, *p* = 0.05, which was not significantly associated with baseline RMSSD as an independent predictor. In contrast to ability EI, total mixed EI was not a significant predictor of baseline RMSSD, *F*(1,99) = 0.26, *p* = 0.61 (Figure 2B). HRV and HR during the baseline resting condition were not significant predictors of EI (Figures 2C–E). See Supplementary Table S1 for RMSSD model coefficients, sums of squares, and partial eta-squared.

#### Physiological Response to Serial Subtraction

A linear mixed model was utilized to assess the response to the serial subtraction task and subsequent recovery following the stress induction. As expected, the model demonstrated that participants showed significant reductions in RMSSD during the stress induction and significant increases during recovery relative to baseline levels, *F*(2,303) = 9.55, *p* < 0.0001. See Supplementary Table S4 for model coefficients, sums of squares, and partial eta-squared.

### EI Predicting Change in CVC Across Conditions

Total ability EI and total mixed EI scores were both incorporated into the physiological response to serial subtraction linear mixed model to assess whether EI could account for changes in RMSSD during stress induction or recovery conditions; beyond the associations observed for RMSSD during the baseline resting condition. There was no significant main effect of total ability EI and RMSSD with condition, *F*(1,301) = 2.03, *p* = 0.08 or total mixed EI and RMSSD with condition, *F*(1,301) = 0.80, *p* = 0.54. No significant interactions between EI and stress induction or recovery conditions were observed for ability EI, *F*(2,294) = 0.86, *p* = 0.42, or mixed EI, *F*(2,294) = 1.95, *p* = 0.14. The addition of total EI scores was not favored over the simpler model only including RMSSD and condition, for the main effect *L.Ratio* = 3.24, *p* = 0.20, or interaction *L.Ratio* = 11.85, *p* = 0.22. See Supplementary Table S5 for model coefficients, standard errors, and beta values.

### Investigating the Potential Influence of Covariates

Gender and caffeine use were tested as potentially influential covariates affecting CVC during rest, based on prior literature (Allen et al., 2007). The combination of both covariates was not favored over the simpler model main effect model only including RMSSD and EI, *nor was their* addition independently. See Supplementary Material S2 for model comparison statistics.

### Exploring Individual Differences in CVC in Response to Stress and Recovery

#### CVC Reactivity Cluster Identification

**FIGURE 3:**
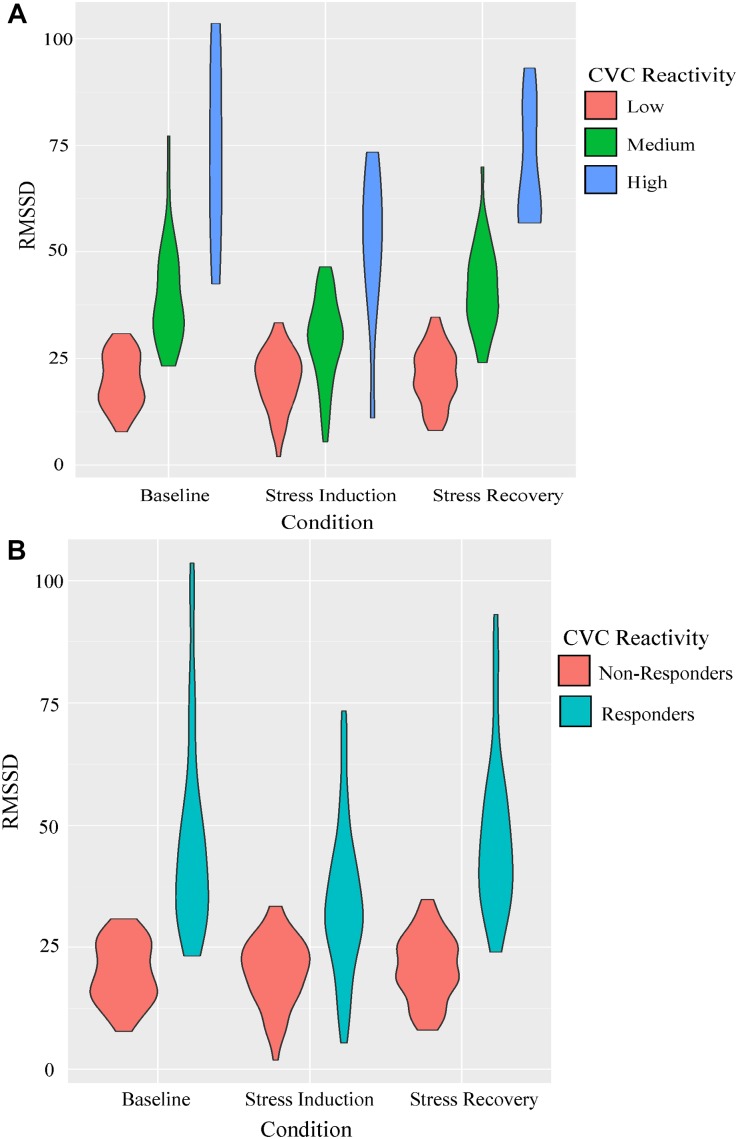
Parameters of RMSSD estimates across conditions for the CVC reactivity groups presented as violin plots. Outlines illustrate kernel probability density, i.e., the width of the shaded area represents the proportion of the data located there. Panel **(A)** represents the initial groupings identified using hierarchical cluster analysis. Panel **(B)** represents the collapsed grouping discriminating CVC reactivity to the stress induction for Mann-Whitney U analyses.

The principal components analysis identified two components that accounted for 97.93% of the cumulative percentage of variance in RMSSD across conditions. Hierarchical agglomerative clustering classified three unique groups based on CVC (Figure 3A). Due to the large variance in the sample sizes between the clusters identified (*n* = 53, *n* = 39, and *n* = 10), the two unique groups that had similar decreases in RMSSD during stress and subsequent increases during the recovery were collapsed to form two groups with comparable sample sizes to assess differences in CVC responsiveness [i.e., CVC-non-responders (*n* = 53) and CVC-responders (*n* = 49)] (Figure 3B). Table 2 presents initial group descriptive statistics, means, and associated standard deviations for the demographic, EI, and physiological variables. Table 3 presents the collapsed groups descriptive statistics, means, and associated standard deviations for the demographic, EI, and physiological variables.

**Table 2:** Descriptive statistics of groups identified using hierarchical agglomerative clustering.

	CVC low responders		CVC middle responders		CVC high responders	
Measure	Mean	St. Dev.	Mean	St. Dev.	Mean	St. Dev.
Age	22.72	4.59	23.16	4.62	21.80	2.10
Baseline RMSSD	19.76	6.21	39.69	11.83	73.44	21.49
Stress induction RMSSD	20.03	6.92	29.49	10.07	51.51	18.15
Stress recovery RMSSD	20.79	6.77	41.42	9.81	70.36	14.19
EQi total	101.21	13.30	105.97	10.83	100.70	13.94
MSCEIT total	104.81	11.59	112.24	13.43	107.44	9.75


*RMSSD, root mean square of successive differences; HRV, heart rate variability; HR, heart rate; EQi, Bar-On EQ-I 2; MSCEIT, Mayer-Salovey-Caruso Emotional Intelligence Test II.*

**Table 3:** Descriptive statistics of the collapsed grouping utilized for group level analyses.

	CVC responders		CVC non-responders	
Measure	Mean	St. Dev.	Mean	St. Dev.
Age	22.88	4.24	22.72	4.59
Baseline RMSSD	46.72	19.76	19.76	6.21
Stress induction RMSSD	34.08	14.99	20.03	6.92
Stress recovery RMSSD	47.45	15.98	20.79	6.77
EQi total	104.88	11.58	101.21	13.30
MSCEIT total	111.24	12.81	104.81	11.59


*RMSSD, root mean square of successive differences; HRV, heart rate variability; HR, heart rate; EQi, Bar-On EQ-I 2; MSCEIT, Mayer-Salovey-Caruso Emotional Intelligence Test II.*

#### CVC Responsiveness to Stress Predicting EI

Compared to CVC-non-responders, individuals that experienced decreases in RMSSD during the stress induction condition and subsequent increases during recovery had significantly higher levels of total ability EI (W = 891, *p =* 0.01). The association was not driven by a specific subscale. There were no significant differences between groups for total mixed EI (W = 1105.5, *p =* 0.26). See Supplementary Table S8 for model coefficients, group means, and standard deviations.

## Discussion

In this study, we investigated the relationship between EI, as measured by two alternate theoretical models, and CVC under resting and reactive conditions. Based on prior findings, we hypothesized that higher ability EI would be associated with higher levels of CVC. Overall, we found support for this hypothesis, although with some qualifications. We discuss these findings and their implications in detail below.

### Primary Hypotheses

First, we hypothesized that individuals with higher levels of ability EI would have greater levels of CVC at rest. This hypothesis was supported, as individuals with higher levels of CVC at rest indeed had significantly higher levels of total ability EI, whereas no association was found with total mixed EI. Contradictory to expectations, higher levels of understanding emotions and the ability to perceive emotions were not significantly associated with resting CVC, even though understanding emotions was found to drive the association with total ability EI. This may be because the ability to perceive emotions is more specific to affective processing and the ability to self-regulate in environments eliciting more personally relevant emotion-specific contexts (Rash and Prkachin, 2013). The only other study investigating ability EI and CVC did not find any association with total EI at rest (Rash and Prkachin, 2013). However, participants in that study may have been aware that the experiment involved a personally relevant experiential sadness induction, as they had to provide a personalized sadness narrative before their laboratory visit. This may have led to affect specific introspective thoughts during the resting period. Our study had greater statistical power than the Rash & Prkachin study and, therefore, may be more representative of the typical resting condition utilized in CVC reactivity assessments (Rash and Prkachin, 2013). Thus, we conclude that individuals with greater demonstrated ability EI, perhaps by those who show a more sophisticated understanding of emotions, the factors that influence them, and how they may evolve over time and during the course of social interactions, also show a greater capacity to regulate cardiac vagal responsiveness.

Second, we hypothesized that individuals who have higher levels of ability EI would have greater decreases in CVC in response to stress and show subsequent increases during recovery. This hypothesis was not supported by initial statistical models. Participants with greater modulation in CVC across the stress induction period (RMSSD decrease) and subsequent recovery (RMSSD increase) did not have higher levels of total ability EI or mixed EI. Baseline differences in CVC appear to drive the observed association with EI since no interactions with stress induction or recovery conditions were found. Although sympathetic and parasympathetic outflows tend to have a close-fitting reciprocal relationship, modes of autonomic control are not always linear (Berntson et al., 1991). Higher resting CVC is associated with situationally appropriate emotional responding and can mitigate the experience of negative emotional arousal in response to stress (Fabes and Eisenberg, 1997). This may represent an adaptive response where activation of parasympathetic systems attenuates sympathetic dominance when individuals experience stressors. From these findings, we conclude that individuals with higher ability EI demonstrate a greater level of resting CVC.

### Exploratory Analyses

As part of the present study, we also sought to clarify how the predictive validity of EI on CVC compares to other cardiac metrics. The observed associations between ability EI and RMSSD, but not HRV or HR, suggest that parasympathetic influences on autonomic control are responsible for the majority of observed associations with ability-based EI. RMSSD is a metric that results in a differential gain function such that it weights more heavily the high-frequency vagal influence whereas HRV is a metric that results in a flat gain function that will capture frequencies that represent a combination of sympathetic and parasympathetic influences (Allen et al., 2007).

We also sought to determine how categorization based on individual differences in CVC reactivity would predict ability EI and mixed EI. Individual categorization based on responsiveness to the stress induction confirmed the relationship observed in the linear regression models. Individuals identified as responders to the stress induction had significantly higher ability EI scores compared to CVC non-responders; while no difference between groups was observed for mixed EI scores. Intriguingly, no specific domain of ability EI displayed significant positive associations with CVC responders. These findings suggest that during stressful experiences, individuals with higher baseline levels of CVC and greater cardiovascular responses, which reflect higher baseline parasympathetic control, greater withdrawal during stressors and greater increases during the subsequent recovery, may have a greater capacity to modulate CVC and in a manner that facilitates the ability to cope with emotional demands.

### Considerations

Our findings are consistent with the only other study that investigated direct associations between HRV as a metric representative of autonomic control and quantified EI (Rash and Prkachin, 2013). Two other studies examining CVC and EI used less known mixed-model-based metrics to quantify EI, which may have contributed to their varied findings, and failure to distinguish associations between EI and baseline CVC (Laborde et al., 2011; Plews et al., 2012). EI as a measurable construct remains highly debated, and the call for an increased focus on refinement in its assessment may lead to greater clarity about the association between EI and CVC (Fiori and Antonakis, 2011). The substantial body of work associating CVC and emotional regulation emphasizes the notion that if EI is validly conceived and measured, at least some aspects should have significant associations with autonomic processes. While the amount of variance accounted for in our significant baseline model using RMSSD (*partial* η^2^ = 0.04) was relatively small, the findings are consistent with the amount of unique variance accounted for by the association between RSA and ability EI in the only other study examining CVC and the MSCEIT (Rash and Prkachin, 2013). The theoretical construct of EI also remains heavily debated, and a multi-level theoretical approach incorporating actual behavioral outcomes will be critical to the construct in achieving its potential for psychometric validity (Boyatzis, 2018). In light of this, we believe that our study offers a unique and valuable insight into the relationship between CVC and EI that will help propel future investigations relating these two constructs (Ioannidis, 2005). Based on the Polyvagal theory, the relationship between higher CVC and reactivity is associated with adaptive and beneficial behavioral responding, which based on the evidence presented, is associated with higher levels of EI. Whether higher CVC leads to higher EI or higher EI results in higher and more responsive CVC is, at present, an empirical question, and one that might be fruitfully examined in a longitudinal developmental study, or in a study training CVC and EI to observe the time-dependent changes in each.

The interplay between emotion and feeling are critical components in the maintenance of health and the facilitation of perception, decision making, and learning; and an inability to integrate the two processes often leads to maladaptive behavior (Damasio, 2001). Decreased CVC is associated with both mild and more severe forms of psychopathology and is becoming a more widely accepted biomarker for susceptibility to emotion dysregulation (Thayer et al., 2012). The cognitive system contributing to autonomic control, as defined by the NVI model, is especially sensitive to negative feedback (Thayer and Lane, 2000). Lower resting CVC is associated with perceived difficulties in emotion regulation, specific to decrements in emotional clarity and impulse control (Williams et al., 2015). Attention regulation and affective processing are necessary to counter sympathetic activation during non-optimal contexts and facilitate social interaction as described by the Polyvagal theory (Porges, 1995). There is also an association between stress-related illness, blunted autonomic regulation, and negative family-of-origin relationship experiences (Luecken et al., 2005). This further highlights the impact of biopsychosocial development on physiologic and emotion regulation capacities that should theoretically relate to the construct of EI. Higher levels of cognitive-emotional abilities contribute to emotion regulation abilities that drive positive behavioral outcomes. A recent systematic review of 135 papers concluded resting CVC is associated with flexible emotional responding and emotion regulation strategies, as well as supports CVC as an objective marker of emotion regulation (Balzarotti et al., 2017). The interplay between decrements in physiological resources (e.g., during sleep deprivation, environmental extremes, emotional stress, and physical hardship), and degradation of cognitive function contribute to potentially detrimental decision making and allude to the need for novel interventions to mitigate the impact of stress on cognitive systems.

The use of biofeedback to augment CVC and its reactivity under stressful conditions is widely used and increasingly has focused on domains ranging from workplace office environments to fitness centers (McCraty et al., 2003; Düking et al., 2017). Interventions targeting emotional processes, such as mindfulness-attention training, can lead to positive outcomes in well-being, and have a substantial impact on emotion-specific neurocognitive processing (Shapiro et al., 2008; Desbordes et al., 2012). Recent work has also demonstrated that EI is malleable and susceptible to increases with targeted training (Alkozei et al., 2018; Mattingly and Kraiger, 2018). The current findings suggest there is a need for further study into the use of training interventions targeting CVC and EI in conjunction as a useful non-pharmacological method for improving well-being; perhaps mitigating symptomology associated with decreases in emotional processing on both an impermanent and pathological level in a manner that promotes well-being.

## Limitations

Several limitations should be considered when interpreting the results of this study. We have interpreted the decrease in RMSSD during stress and subsequent increase during recovery as evidence of an optimal adaptive emotion regulation process, relative to the experience of stress. While the stress induction indeed produced a significant decrease in CVC across the sample of participants, a subset of individuals experienced increases in autonomic control during the stress induction or no change at all. It is possible that some individuals did not take the task seriously and did not actively engage in the serial subtraction task. Using a multi-faceted stress induction, such as the serial subtraction task in conjunction with a cold pressor or the Trier Social Stress Test, as well as variations in stressors more specific to different emotions may be more appropriate in future work to assess the relationship between EI and the experience of stress. The use of an affect induction, such as sadness, would also provide valuable information on individual differences in the associations between CVC, stress, affect, and EI.

Recent work has demonstrated the usual reciprocal relationship between the sympathetic and parasympathetic systems, representing the widely accepted fluctuations in autonomic control in response to stress, is dependent on individuals’ cumulative exposure to risk, and resting sympathetic activation (Giuliano et al., 2017). It is possible that the exposure to stress and adversity may have moderated the response to the serial subtraction task and contributed to the observed individual differences in CVC. Further exploration of the influence of cumulative life experiences on CVC and EI is necessary. Of note, we did not collect data on body mass index or specific to anxiety/depression in the present study, which have both been shown to impact HRV. It is conceivable that these unmeasured factors may have also influenced the statistical models and would be appropriate to examine in future work. Participants also completed EI assessment measures after the stress reactivity assessment, leaving the potential for the residual effects of the stress induction to affect individual’s performance on the subsequent measurement of EI, and measurements assessing the behavioral level of EI were also not collected. Lastly, multiple biological systems and factors influence autonomic control, and the associations observed between EI and CVC should not be taken to indicate a causal connection (Bauman et al., 2002).

## Conclusion

The present study examined the association between the mixed and ability models of EI and their relation to CVC at rest and in response to a stressor. These findings help clarify the relationship between individual differences in the two most widely used metrics of EI for the mixed and ability models and their associations with CVC. Higher levels of total ability EI and the ability to understand the complexities of emotions were associated with an index of cardiac parasympathetic control at rest. Larger reductions in parasympathetic control during stress and the ability to recover were also found to be associated with higher total ability EI and driven by the ability to understand emotions. These results suggest that differences in the ability to understand emotional processes in oneself and reason about one’s visceral experience may facilitate better cognitive and emotional processing. Additional research is needed to clarify the degree to which affect influences the relationship between stress and EI, as well as whether improvements in EI can also lead to subsequent increases in CVC or vice versa.

## Ethics Statement

This study was carried out in accordance with the recommendations of the Institutional Review Board (IRB) of the University of Arizona and the United States Army Human Research Protection Office (HRPO). All subjects gave written informed consent in accordance with the Declaration of Helsinki. The protocol was approved by the Institutional Review Board (IRB) of the University of Arizona and the United States Army Human Research Protection Office (HRPO).

## Author Contributions

JV analyzed the data and wrote the initial draft of the manuscript. AA contributed to writing the initial draft of the manuscript. AR contributed to the statistical analysis and writing of the manuscript. JA aided in study design, as well as, contributed to data processing, statistical analysis, and writing of the manuscript. WK designed and supervised all aspects of the study and contributed to the writing of the manuscript.

## Conflict of Interest Statement

The authors declare that the research was conducted in the absence of any commercial or financial relationships that could be construed as a potential conflict of interest.

## Footnotes

1 https://www.zephyranywhere.com/media/download/zephyr-performance-biopatch-hp-brochure.pdf

2 https://pstnet.com/products/e-prime/

3 https://www.mathworks.com

4 https://www.r-project.org
