# Supplementary material for: Ability-Based Emotional Intelligence Is Associated With Greater Cardiac Vagal Control and Reactivity
Source: Front Hum Neurosci. 2019 Jun 11;13:181. doi: 10.3389/fnhum.2019.00181 (PMC6579931; doi:10.3389/fnhum.2019.00181)
Supplement: Supplementary file 1 [file Table_1.pdf]

Appendix A. List of all medications used regarding exclusion criterion in the study

**Medications (used as an exclusion criterion)**	**Mechanism/Cardiac Affect**
Advair	Beta-2 Agonist
Allegra	Antihistamine
Atomoxetine	Selective Norepinephrine Reuptake Inhibitor
Claritin	Antihistamine
Lamotrigine	Anticonvulsant- QRS Prolongation
Levothyroxine	Thyroid Hormone
Methotrexate	Antimetabolite Antineoplastic Agent
Ritalin	Central Nervous System Stimulant
Suboxone	Opioid Agonist
Trazadone	Alpha-1 Antagonist
Wellbutrin	Dopaminergic Antagonist
Zyrtec	Antihistamine

**Medications (not used as an exclusion criterion)**	**Mechanism/Use**
Amethia	Birth Control
Citalopram	Selective Serotonin Reuptake Inhibitor
Escitalopram	Selective Serotonin Reuptake Inhibitor
Kariva	Birth Control
Microgestin	Birth Control
Norethindrone	Birth Control
Sertraline	Selective Serotonin Reuptake Inhibitor
