# Supplementary material for: Ability-Based Emotional Intelligence Is Associated With Greater Cardiac Vagal Control and Reactivity
Source: Front Hum Neurosci. 2019 Jun 11;13:181. doi: 10.3389/fnhum.2019.00181 (PMC6579931; doi:10.3389/fnhum.2019.00181)
Supplement: Supplementary file 2 [file Table_2.pdf]

S.1 EI, HRV, and HR

Neither ability EI nor mixed EI were significant predictors of baseline HRV or HR (Figure 2, Panels C-F). See Table S2 for HRV model coefficients, sums of squares, and partial eta-squared and Table S3 for HR model coefficients, sums of squares, and partial eta-squared.

Physiological Response to Serial Subtraction

A linear mixed model established that participants did not have significant changes in HRV during the stress reactivity assessment, $F(2, 300) = 2.45$, $p = .09$. An additional linear mixed model indicated that participants did have significant increases in HR in response to the stress induction and no change relative to baseline levels during recovery, $F(2, 300) = 81.59$, $p < .0001$. See Table S4 for model coefficients, standard errors, and beta values.

EI Predicting Change Across Conditions

No interactions or main effects for EI and HRV across conditions were observed for any EI measures of interest. See Table S6 for HRV model coefficients, standard errors, and beta values. There were also no interactions or main effects for EI and HR across conditions. See Table S7 for HR model coefficients, standard errors, and beta values.

S.2 Investigating the Potential Influence of Covariates

The combination of gender and caffeine was not favored over the simpler model main effect model only including RMSSD and EI, *L.Ratio* = 0.46, $p = .79$. The addition of gender independently, *L.Ratio* = 0.03, $p = .86$, or caffeine used, *L.Ratio* = 0.42, $p = .52$, were also not

favored over the simpler main effects models when interrogated on their inclusion. The combination of both covariates was also not favored over the simpler model main effect model only including HRV and EI, *L.Ratio* = 1.65, $p = .44$. The addition of gender independently, *L.Ratio* = 0.76, $p = .38$, or caffeine used, *L.Ratio* = 0.80, $p = .37$, were also not favored over the simpler main effects models when interrogated on their inclusion. The combination of both covariates was not favored over the simpler model main effect model only including HR and EI, *L.Ratio* = 1.42, $p = .49$. The addition of gender independently, *L.Ratio* = 1.34, $p = .25$, or caffeine used, *L.Ratio* = 0.11, $p = .74$, were also not favored over the simpler main effects models when interrogated on their inclusion.

S.3 Exploring Individual Differences in CVC in Response to Stress and Recovery

Zero-Order Subscale Correlation Analysis

Bivariate correlations assessing relationships among RMSSD, HRV, and HR at baseline resting levels, change from the prior level during stress induction and during resting recovery, MSCEIT subscale scores, and EQi subscale scores across the total sample. The MSCEIT understanding branch showed positive associations with baseline RMSSD and negative associations with change in RMSSD from baseline to stress induction. The MSCEIT managing branch showed positive associations with change in RMSSD from stress induction to resting recovery. The EQi decision-making branch and self-expression branch showed negative associations with change in RMSSD from baseline to stress induction. The EQi decision-making branch and self-perception branch showed positive associations with change in RMSSD from stress induction to resting recovery. None of the observed associations remained significant after Bonferroni correction for

multiple comparisons. See Figure S.1 for Spearman correlation coefficients with HRV and with HR.

Table S1. EI Scores Predicting RMSSD at Rest Linear Models

		**Baseline RMSSD During Rest**			
Predictors	*SS*	*df*	*F*	*p*	*Partial* η^2
EQI Total	0.08	1	0.26	0.61	0.003
MSCEIT Total	1.37	1	4.60	0.03	0.044
Residuals	29.41	99			
MSCEIT Understanding Branch	1.16	1	3.89	0.05	0.037
Residuals	29.70	100			

RMSSD: root mean square of successive differences; EQI: Bar-On EQ-I; MSCEIT: Mayer-Salovey-Caruso Emotional Intelligence Test II

Table S2. EI Scores Predicting HRV at Rest Linear Models

	**Baseline HRV During Rest**				
Predictors	*SS*	*df*	*F*	*p*	*Partial* η^2
EQI Total	0.00	1	0.05	0.82	0.001
MSCEIT Total	0.49	1	2.71	0.10	0.027
Residuals	1.80	99			

HRV: Heart Rate Variability; EQI: Bar-On EQ-I; MSCEIT: Mayer-Salovey-Caruso Emotional Intelligence Test II

Table S3. EI Scores Predicting HR at Rest Linear Models

Baseline HR During Rest					
Predictors	*SS*	*df*	*F*	*p*	*Partial* η^2
EQI Total	0.03	1	1.87	0.17	0.019
MSCEIT Total	0.01	1	0.79	0.38	0.008
Residuals	1.69	99			

RMSSD: root mean square of successive differences; EQI: Bar-On EQ-I; MSCEIT: Mayer-Salovey-Caruso Emotional Intelligence Test II

Table S4. Physiological Response to Serial Subtraction

	**RMSSD**			
Predictors	*Estimates*	*std. Error*	*Statistic*	*p*
(Intercept)	28.07	0.05	60.83	**<0.001**
Stress Induction	-0.84	0.06	-3.05	**0.002**
Stress Recovery	1.04	0.02	2.03	**0.043**
	**HRV**			
Predictors	*Estimates*	*std. Error*	*Statistic*	*p*
(Intercept)	7.19	0.01	154.14	**<0.001**
Stress Induction	1.03	0.01	2.21	**0.028**
Stress Recovery	1.01	0.01	1.22	0.223
	**HR**			
Predictors	*Estimates*	*std. Error*	*Statistic*	*p*
(Intercept)	83.91	0.01	324.29	**<0.001**
Stress Induction	1.12	0.01	10.2	**<0.001**
Stress Recovery	0.98	0	-3.98	**<0.001**
Observations	306			

Table S5. EI Predicting Change in RMSSD Across Conditions

Predictors	*Estimates*	*std. Error*	*Statistic*	*p*	*Estimates*	*std. Error*	*Statistic*	*p*
(Intercept)	28.07	0.05	61.08	**<0.001**	28.03	0.05	60.78	**<0.001**
RMSSD Stress Induction:	0.84	0.06	-3.05	**0.003**	0.84	0.06	-2.94	**0.004**
RMSSD Recovery After Stress:	1.04	0.02	2.03	**0.044**	1.04	0.02	1.91	0.057
EQi Total	0.8	0.37	-0.61	0.541	1.24	0.44	0.48	0.63
MSCEIT Total	2.03	0.41	1.74	0.082	2.68	0.48	2.05	**0.041**
RMSSD Stress Induction: EQi Total					0.43	0.47	-1.79	0.075
RMSSD Recovery After Stress: EQi Total					0.93	0.17	-0.44	0.66
RMSSD Stress Induction: MSCEIT Total					0.57	0.51	-1.11	0.267
RMSSD Recovery After Stress: MSCEIT Total					0.94	0.18	-0.35	0.727
Observations	306				306			

Table S6. EI Predicting Change in HRV Across Conditions

Predictors	*Estimates*	*std. Error*	*Statistic*	*p*	*Estimates*	*std. Error*	*Statistic*	*p*
(Intercept)	7.19	0.01	154.17	**<0.001**	7.19	0.01	152.53	**<0.001**
HRV Stress Induction:	1.03	0.01	2.21	**0.028**	1.03	0.01	2.25	**0.025**
HRV Recovery After Stress:	1.01	0.01	1.22	0.223	1.01	0.01	1.14	0.253
EQi Total	0.96	0.08	-0.47	0.638	1.03	0.1	0.25	0.802
MSCEIT Total	1.14	0.09	1.39	0.164	1.21	0.11	1.66	0.098
HRV Stress Induction: EQi Total					0.89	0.12	-0.98	0.328
HRV Recovery After Stress: EQi Total					0.97	0.05	-0.67	0.506
HRV Stress Induction: MSCEIT Total					0.88	0.13	-0.94	0.346
HRV Recovery After Stress: MSCEIT Total					0.99	0.06	-0.26	0.794
Observations	306				306			

Table S7. EI Predicting Change in HR Across Conditions

Predictors	*Estimates*	*std. Error*	*Statistic*	*p*	*Estimates*	*std. Error*	*Statistic*	*p*
(Intercept)	83.91	0.01	322.15	**<0.001**	83.93	0.01	319.14	**<0.001**
HR Stress Induction:	1.12	0.01	10.2	**<0.001**	1.12	0.01	10.22	**<0.001**
HR Recovery After Stress:	0.98	0	-3.98	**<0.001**	0.98	0	-3.81	**<0.001**
EQi Total	0.95	0.1	-0.54	0.587	0.87	0.11	-1.26	0.208
MSCEIT Total	0.97	0.11	-0.24	0.809	0.92	0.12	-0.66	0.508
HR Stress Induction: EQi Total					1.19	0.09	1.88	0.061
HR Recovery After Stress: EQi Total					1.04	0.03	1	0.318
HR Stress Induction: MSCEIT Total					1.12	0.1	1.11	0.269
HR Recovery After Stress: MSCEIT Total					1	0.04	-0.04	0.97
Observations	306				306			

Table S8. CVC Responsiveness Predicting EI Mann-Whitney U Test Models

Predictors	*W*	*p*	*Mean*	*SD*
EQI Total	1105.5	.259		
Non-CVC-Responders			101.21	13.30
CVC-Responders			104.88	11.58
MSCEIT Total	891	**.010**		
Non-CVC-Responders			104.81	11.59
CVC-Responders			111.24	12.81
MSCEIT Understanding	997	.062		
Non-CVC-Responders			108.42	18.42
CVC-Responders			115.37	19.10
MSCEIT Managing	1097	.235		
Non-CVC-Responders			99.55	12.27
CVC-Responders			102.40	11.25
MSCEIT Perceiving	945	.026		
Non-CVC-Responders			107.28	8.47
CVC-Responders			113.49	15.86
MSCEIT Using	1042	.119		
Non-CVC-Responders			104.72	13.25
CVC-Responders			109.12	13.52

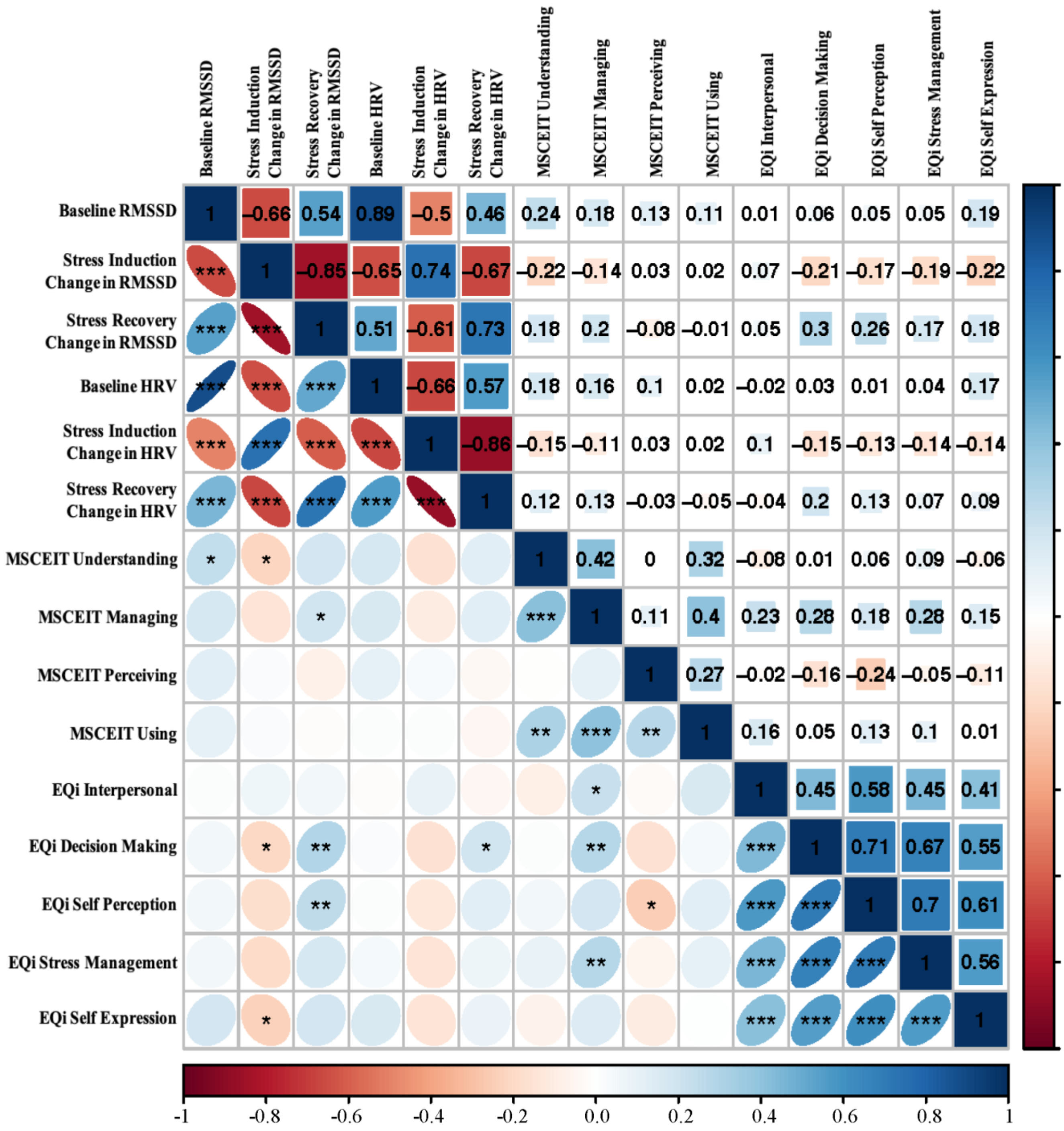

Figure S1. Bivariate Spearman correlations performed across all subjects with correlation coefficients in the upper portion of the matrix and significant correlations identified in the lower portion of the matrix. * p<.05, ** p<.01, *** p<.001 RMSSD: root mean square of successive differences; HRV: heart rate variability; EQi: Bar-On EQ-I 2; MSCEIT: Mayer-Salovey-Caruso Emotional Intelligence Test II
